# Voluminous Seminal Vesicle Cyst Treated by Laparoscopic‐Guided Alcoholization: A Case Report and Review of the Literature

**DOI:** 10.1155/criu/8835546

**Published:** 2026-10-05

**Authors:** Giuseppe Pizzuto, Paolo Massenio, Vincenzo Stellacci, Francesco Lafranceschina, Salvatore Giordano, Felice Arborea, Fabio Ambruoso, Andrea Capurso, Mario De Siati

**Affiliations:** ^1^ Ospedale Della Murgia “Fabio Perinei” ASL Bari Altamura Italy

**Keywords:** alcohol sclerotherapy, laparoscopy, lower urinary tract symptoms, seminal vesicle cyst, Zinner syndrome

## Abstract

Seminal vesicle cysts are uncommon anomalies of the male genitourinary tract and are often discovered incidentally. When large, they may become symptomatic, causing lower urinary tract symptoms and hematospermia. Surgical excision is generally recommended for symptomatic cases, although this approach may be associated with nonnegligible morbidity. We report the case of a large seminal vesicle cyst associated with ipsilateral renal agenesis, successfully treated with laparoscopic‐guided aspiration and alcohol sclerotherapy. To our knowledge, this technique has not previously been described. The procedure resulted in symptom resolution and radiological disappearance of the cyst at short‐term follow‐up.

## 1. Introduction

Cysts of the seminal vesicles are rare entities that may be either congenital or acquired. In most cases, they remain asymptomatic and are detected incidentally during imaging studies performed for unrelated reasons [1, 2]. However, when cysts increase in size, they may lead to clinically relevant symptoms, including hematospermia, dysuria, urinary urgency, urinary retention, ejaculatory disorders, or infertility [3, 4].

Congenital seminal vesicle cysts are frequently associated with abnormalities of the mesonephric (Wolffian) duct. As this embryological structure gives rise to the kidney, ureter, vas deferens, epididymis, and seminal vesicle, an early developmental alteration may result in ipsilateral renal agenesis and obstruction of seminal vesicle outflow [5, 6]. This association, known as Zinner syndrome, represents a rare congenital condition that is typically diagnosed in adulthood [7].

Magnetic resonance imaging (MRI) plays a central role in diagnosis, allowing accurate anatomical assessment and reliable exclusion of malignant features [8, 9]. Treatment is generally reserved for symptomatic patients. Open, laparoscopic, and robot‐assisted excision are the most commonly reported approaches; however, these procedures involve removal of the cyst and often of the seminal vesicle itself, with a potential risk of injury to adjacent pelvic organs. In this report, we describe a minimally invasive alternative based on laparoscopic‐guided aspiration and alcohol sclerotherapy of a large seminal vesicle cyst.

## 2. Case Presentation

A 44‐year‐old man presented with progressive lower urinary tract symptoms, including strangury, dysuria, and urinary urgency, which had gradually worsened over approximately 18 months. He also reported recurrent episodes of hematospermia. Medical therapy with alpha‐blockers did not provide symptomatic relief.

Abdominal ultrasound revealed a large cystic pelvic lesion. Subsequent pelvic MRI demonstrated a well‐defined cyst arising from the right seminal vesicle, measuring approximately 10 × 5 × 11 cm (Figures 1 and 2). The lesion was hypointense on T1‐weighted images and hyperintense on T2‐weighted images, with no enhancement after contrast administration, findings consistent with a benign cystic lesion.

**Figure 1:**
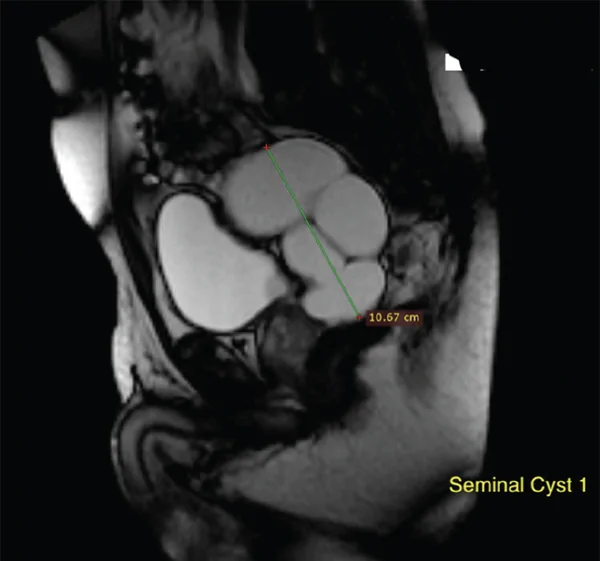
Sagittal diameter of the seminal vesicle cyst.

**Figure 2:**
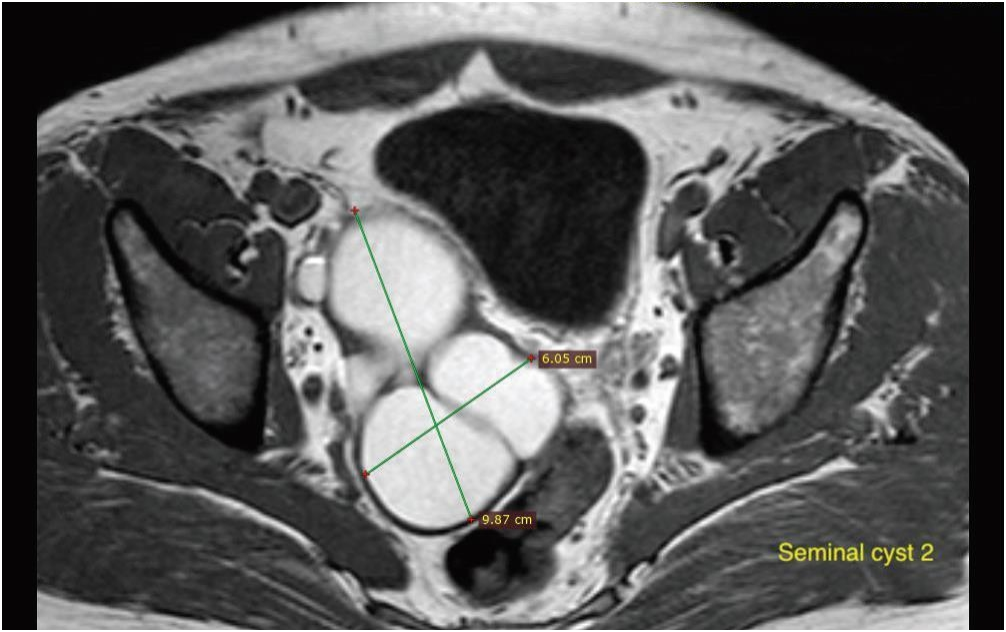
Longitudinal diameter of the seminal vesicle cyst.

The cyst exerted a significant compressive effect on the urinary bladder, which correlated with the patient′s urinary symptoms (Figure 3). MRI also demonstrated right renal agenesis, hypotrophy of the right testicle, and absence of the epididymis, findings consistent with Zinner syndrome. Cystoscopy was performed and excluded urethral strictures, fistulas, or intravesical pathology. Given the close proximity of the cyst to the internal iliac vessels and the colon, a percutaneous interventional radiology‐guided approach was considered unsafe.

**Figure 3:**
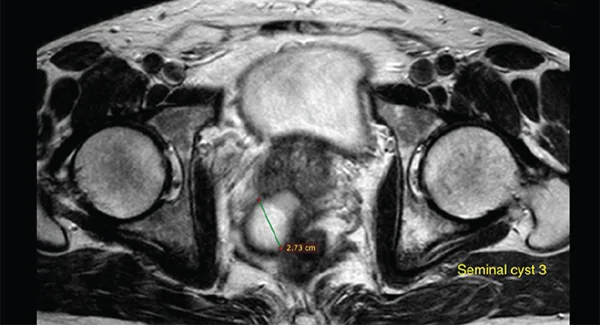
Seminal vesicle cyst and prostate.

## 3. Surgical Technique

A laparoscopic approach was selected to allow direct visualization of the pelvic anatomy and to reduce the risk of injury to surrounding structures. A 12‐mm Hasson trocar was placed at the hypogastric level along the pubic‐umbilical line, and a 5‐mm trocar was positioned in the left iliac fossa. After entering the retropubic space (space of Retzius), the cyst was immediately identified without the need for extensive dissection.

Under laparoscopic control, a 12Ch mono‐J catheter was introduced into the cyst using a Chiba needle over a guidewire. Approximately 200 mL of serohematic fluid was aspirated. Iodinated contrast medium was then injected into the cyst to exclude leakage or communication with adjacent organs. Subsequently, methylene blue was instilled with simultaneous cystoscopy to rule out communication with the urinary tract.

After complete aspiration of the injected fluids, 50 mL of 96% ethanol was instilled into the cystic cavity and left in place for approximately 15 min before being aspirated. The mono‐J catheter was left in situ, and a tubular drain was placed through the left iliac fossa port. The drain was removed on the first postoperative day. Due to persistent minimal drainage from the mono‐J catheter, a second alcohol sclerotherapy session was performed on postoperative day 2 using the same technique. The patient was subsequently discharged, and the mono‐J catheter was removed on postoperative day 10 after complete cessation of drainage.

## 4. Follow‐Up

Four months after the procedure, contrast‐enhanced CT imaging demonstrated complete resolution of the cystic cavity, which had been replaced by fibrotic tissue (Figures 4, 5, and 6). Clinically, the patient reported complete resolution of hematospermia and a marked improvement in urinary symptoms.

**Figure 4:**
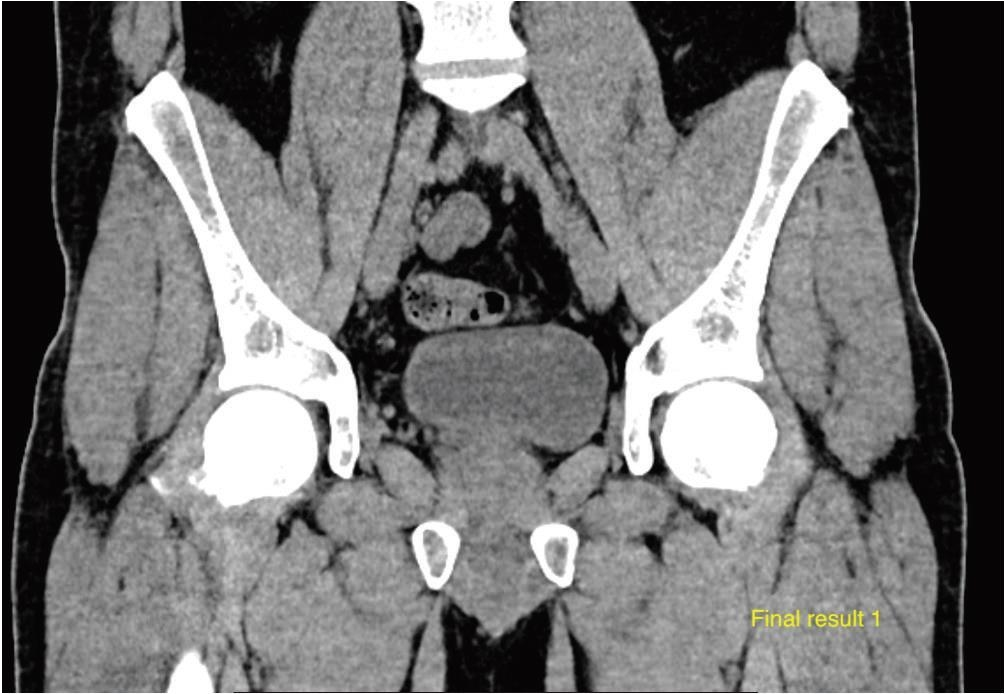
Coronal view of the pelvis after surgical treatment.

**Figure 5:**
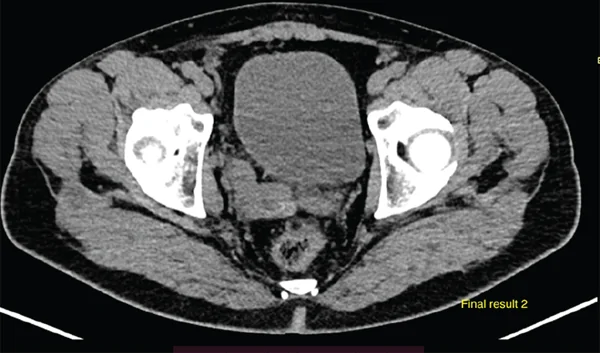
Longitudinal view of the pelvis after surgical treatment.

**Figure 6:**
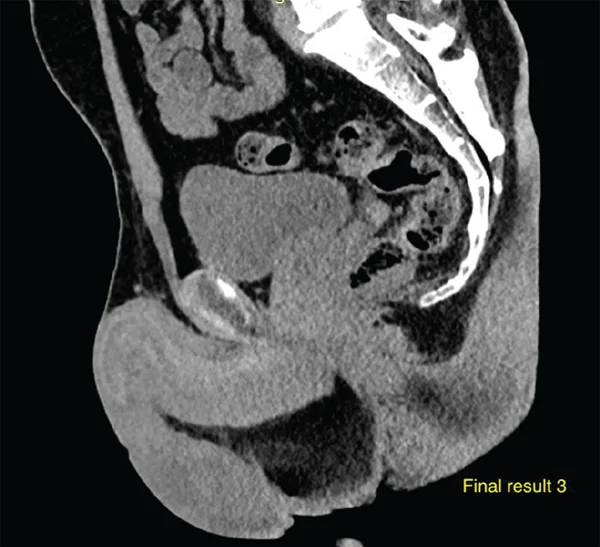
Sagittal view of the pelvis after surgical treatment.

## 5. Discussion

Seminal vesicle cysts are uncommon, and their pathogenesis is closely related to developmental abnormalities of the mesonephric duct. Obstruction of seminal vesicle drainage, combined with persistent epithelial secretion, leads to gradual cyst enlargement and symptom progression. This mechanism also explains the limited efficacy of simple aspiration, which does not eliminate the secretory epithelium and is therefore associated with a high risk of recurrence [10].

Surgical excision remains the most commonly reported treatment for symptomatic cysts and can be performed through open, laparoscopic, or robot‐assisted approaches [11–13]. Although effective, these procedures are invasive and may be associated with complications such as rectal or bladder injury, neurovascular damage, and ejaculatory dysfunction.

Alcohol sclerotherapy following cyst aspiration has been successfully used in the treatment of renal, hepatic, and breast cysts, where it induces chemical ablation of the epithelial lining and subsequent fibrosis [14–17]. To date, its application to seminal vesicle cysts has not been described. In the present case, laparoscopic guidance allowed safe alcoholization of the cyst while preserving surrounding anatomical structures and avoiding cyst excision. The main limitation of this report is the relatively short follow‐up period, which does not allow definitive conclusions regarding long‐term recurrence. Nevertheless, the favorable clinical and radiological outcome suggests that this minimally invasive approach may represent a valid alternative in selected patients.

## 6. Conclusions

Laparoscopic‐guided aspiration and alcohol sclerotherapy may represent a feasible and less invasive alternative for the treatment of large symptomatic seminal vesicle cysts, provided that malignancy and communication with the urinary tract have been excluded. Further experience and longer follow‐up are required to confirm the long‐term efficacy of this technique.

## Author Contributions

Dr. Giuseppe Pizzuto was the writer and creator of the article. Dr. Paolo Massenio, Dr. Vincenzo Stellacci, Dr. Francesco Lafranceschina, Dr. Salvatore Giordano, Dr. Felice Arborea, Dr. Fabio Ambruoso, and Dr. Andrea Capurso have cooperated in data collection. Dr. Mario De Siati cooperated and was the supervisor and coordinator of the project.

## Funding

No funding was received for this study.

## Ethics Statement

The clinical case was managed in accordance with the principles of good clinical and ethical practice. Written informed consent was obtained from the patient.

## Consent

All the patients allowed personal data processing, and informed consent was obtained from all individual participants included in the study.

## Conflicts of Interest

The authors declare no conflicts of interest.

## Data Availability

Clinical documentation and imaging were retrieved during hospitalization and subsequent follow‐up visits after obtaining informed consent from the patient.
